# An Ishmaelite

**Published:** 1888-05-19

**Authors:** Leslie Keith

**Affiliations:** Author of "The Chilcotes," "Uncle Bob's Niece," "East and West," etc.


### AN ISHMAELITE.

By Leslie Keith,

Author of "The Chilcotes," "Uncle Bob's Niece," "East and West," etc.

### Chapter VII.—The New Housekeeper.

The arrangement that placed the reins of household management in the hands of Ella Coates, and made her virtually mistress of Mr. Eastgate's establishment, was the outcome of a little successful diplomacy on cousin Fanny's part.

It had not been found possible to leave Ella in possession on that first visit at the time of Helen Eastgate's death, even so clever a strategist as Fanny could not see her way to that successful move, so Ella and the big box—filled with some of the poor dead lady's possessions—were trundled back to the vicarage. On the occasion of Nellie's marriage, however, the indomitable Fanny saw her opportunity.

Nellie's wedding took place in less than a year after her aunt's death. The girl had pined and fretted, and grown thin and sad in the gaunt, big house whence all the brightness had long ago departed, and, naturally enough, the first aspirant who appeared took in her eyes the light of a deliverer. Nellie did not pause to ask herself if she cared for this big, good-humoured champion, whom she had known in happier days, and who now came to rescue her from imprisonment. It was enough to know that he liked her. Her little heart had been bruised with too heavy a weight for it to bear, and she found a refuge in this careless affection that asked so little.

Her uncle gave her and her fortune willingly enough into the hands of a man who had a good name in the City, and private means as well. He had never cared for Nellie, and was not sorry to be rid of her. Her white face, her big, wistful eyes, and her timid, abashed ways, reminded him, too uncomfortably for his peace, of his wife, and of the son whom he had disowned. Fred's eyes, too, had had a way of dropping before his angry gaze; and there were moments when he almost hated Nellie for the memories she awoke in him.

When rumour of Nellie's betrothal sped to Norfolk, Fanny made all her plans.

"You may order yourself two new gowns from Miss Lamb, Ella, and I will ask papa if he will let you get a bonnet from Madame Blanc's. I am going to take you to town with me."

The placid Ella obeyed with cheerful alacrity. Two new frocks at once had never fallen to her lot before; and all the younger troop of girls sighed and envied. They had to be content with last year's finery, or with some of poor cousin Helen's spoils made over; but the reverend Fanny, as she was sometimes called by irreverent outsiders, permitted no grumbling.

"Ella will see it to be her duty to help you by-and-by," and Ella knew quite well, without a whisper of her mother's intentions falling on her ear, that she was expected to marry well, and scheme for the younger brood.

Fanny naturally enough wanted to do the best for her children, of whom she had a very full quiver. The vicar had long ceased to expect preferment or perhaps to wish it, and he droned on at the same old sermons week by week and year by year, to the entire contentment of himself and his parishioners, who held that the sermon was chiefly useful as a sedative. If, mated to a husband of this easy-going order, Fanny did not bestir herself, what was to become of the boys and girls? Fanny wrote to Cousin Joseph that she was coming to be a mother to Nellie, and to support her on the trying occasion.

Even Joseph smiled over this conventional expression. "Fanny would willingly enough expose her own six girls to a similar trial," he said. But it was certain he would have allowed no one else to laugh at her. Fanny was a sensible woman, and she was on his side. He wouldn't have any of the Blackrock lot coming to make a fuss over the girl. Fortunately she had no kindred on her own mother's side to take the office Fanny proposed to fill.

When the ceremony was over (the late family bereavement allowed it to be very quiet), Joseph was will·ng enough to put Ella in Nellie's place as housekeeper.

Fanny had discovered in the course of her investigations that the cook sold the dripping, and that other enormities went on in the lower regions. She had a very healthy contempt for Nellie's management.

"She was sadly, sadly, imposed upon," she said, "but Ella will not permit herself to be taken advantage of by the servants. I have trained the dear girl to be useful and exact. Bring your book and let cousin Joseph see how neatly you keep your own little accounts, my dear."

Ella brought the book wherein all her small expenses were duly set forth and carried over and balanced to a farthing. The two new gowns made the biggest item, but there were also sundry threepenny pieces recorded as dropped into the bag on Sunday.

Mr. Eastgate growled out his approval.

"You may stop for a month and see how we get on," he said. "If you have inherited your mother's good sense you will do."

By the end of the month nothing was said about Ella's departure, and the fourth anniversary of Mrs. Eastgate's death found her still in possession. She was all that her mother had promised. Neat, punctual, an able caterer. The table had never been so good, the house so clean, the service so methodical; the machinery worked with perfect smoothness under her reign, and Arthur and his uncle had never had their comforts so well attended to. She was pleasant to look upon too; generously developed, fair and rosy, and so placid and unemotional that nothing ever seemed to ruffle or disturb her. It was not until you knew her very well indeed that you discovered what an inflexible will lay under this composed and pleasant exterior.

Arthur did not find it out till he had secured the right to call all those pleasing qualities his own.

How did this come about? How do these things ever come about? Arthur was generous and chivalrous towards every woman, and disposed to like them all; he saw more of this one than he did of any other; she played to him in her neat, smooth way after dinner in the drawing-room, she sewed the buttons on his shirts and darned his socks, and listened while he talked, and finally, when he asked her the great question, she said "Yes" with a very becoming and modest blush.

Mr. Eastgate sanctioned the engagement; if he was not hearty over it he was not hearty over anything nowadays.

"You've the money," he said, "but she has sense, and that counts for everything in marriage." He was secretly pleased that Arthur had chosen a wife from his side, and he gave Ella a cheque on the spot to get herself some new clothes.

It was over the subject of Fred that Arthur made the discovery of the rock that underlay Ella's amiability. Arthur had talked to her often enough of his cousin, and she had seemed to listen with sympathy, but when he announced his intention of going abroad with Fred and making a new home somewhere across the seas, Ella spoke out her mind.

"I think that would be very foolish," she said.

"Why foolish? One can live as comfortably there as here, and the poor fellow would find it harder to begin again here."

"That would be out of the question. But why can't he begin out there alone?"

"Because I don't intend he should," said Arthur, growing nettled and speaking with determination. "I mean to go with him; I told you so long ago, Ella. Do you mean to say you have changed your mind about going with me?"

"I don't see how you could expect me to go, Arthur," said Ella, going on placidly working. "Why should I give up my country and my friends for the sake of a young man who has been a sorrow and a disgrace to everybody connected with him? When people sin they must expect to suffer for it."

"Oh, how bitterly cruel you women can be," said Arthur. "How righteous, how holy! Do you never sin or fall yourselves, or are you beyond the need of help and pardon?"

"I am not cruel, Arthur," interposed Ella with unruffled calm. "You are talking very unwisely; I am only sensible."

"Ah, very sensible."

"I do not think you are showing me much consideration, Arthur. Why should I be banished for the sake of a whim on your part? I always expected to have a house in London, perhaps to live here, where we could both take care of your uncle; certainly not to be carried off to the other side of the world, and to have the society of a person I cannot respect thrust upon me."

"I am very sorry to disappoint your expectations," said Arthur with more sadness than bitterness, "but I cannot change. I gave my solemn promise before I had ever seen you, and I will keep it at any cost."

"I don't say you shouldn't help to give your cousin a new start if you wish to do so, though I think help in such cases is usually thrown away," said Ella, in that tone of calm reproof that can be so frightfully irritating. "But you could give him money —"

"Yes, and ship him off, and thank Heaven that Australia is six weeks' distant at the nearest, and that poor devils of prodigals sent out there have usually no money to come back with. I love my cousin, Ella, I loved him before I loved you —"

Perhaps he was cruel, inconsiderate, as she said, but her pulse beat no faster than usual even when he banged out of the room in a rage. She was not afraid of this ridiculous scheme. Arthur was so impulsive, but he would presently see how foolish it was; if necessary, mamma could be summoned from Norfolk, and she and cousin Joseph together would soon bring him to reason.

She thought no more of the matter, but presently fell to considering what alterations she should make in the drawing-room if she came back as a bride to Portland Place, and how she should have new chair-covers, and perhaps a new carpet. The velvet pile was certainly a little worn near the windows.

But if Arthur's resolution had required strengthening, she had taken the best possible way to give it backbone. Strong-willed people like Ella sometimes lose everything by refusing to yield in trifles. If she had shown a little sympathy, Arthur might have been more gentle. But her coldness hardened him. It was on the following day that he paid that visit to Nellie that has been recorded, and he went home from Pont Street determined to speak to his uncle and tell him of his plans.

This was the most difficult task that Arthur had yet undertaken in that generous enterprise on which he had set his heart. For years no one had named Fred's name in the merchant's hearing, and though he had resumed his former habits, and went to solemn, pompous dinners, and gave them too, it was as a widower and childless he appeared before the world.

"Uncle Joseph," said Arthur, when the two sat over their wine in the big, grim dining-room, "you remember when you were good enough to offer me a share as junior partner in the house, I refused the honour because ——"

"I thought you a precious fool for refusing."

Arthur passed over this growling interruption.

"I refused because, as I told you, I might have to go on a journey soon, and I wished to hold myself free for what might turn up. The time has come now; I must start to-morrow."

"You are your own master, I suppose. Do you want my permission?" said the elder. "You've money enough to squander in travel, if that's your fancy. You turned up your nose at the business your father helped to make, and thought yourself too fine for it; but Eastgate and Moore managed to pull along without you. If it's the money you put in it you want to withdraw, you can take it and welcome; the loss of it won't break us." He spoke rapidly and angrily, perhaps in his heart of hearts divining what was coming.

"I don't want the money yet," said Arthur, in a low voice. "The interest is more than I can spend. If I should want the capital"—he looked at the gloomy face at the other side of the table and stopped precipitately. Then he said, with a sort of pained abruptness:

"I am going with Fred across the seas—to begin over there."

Mr. Eastgate looked at Arthur, with a terrible anger suddenly flashing over his dark face. The veins on his forehead were swelled, his cheeks and lips were livid.

"Do you dare to mention that—that felon in my hearing," he began.

Arthur started up too, upsetting his wineglass. Very high words, and perhaps an irreconcilable quarrel might have followed, but at that instant the butler announced the staring and wondering Mr. Mack.

"What is it, Arthur, my boy, what is it, eh?" he said, looking from one angry face to the other.

Arthur turned round with sad and wrathful looks.

"My cousin comes out of gaol to-morrow, after five years—five years—gracious heavens! can any of us understand what these years have been to him? And—and his father disowns him! But I am going with him, I am going with Fred; and he will lose me too. He may turn me out too if he will."

Mr. Mack looked from the uncle to the nephew and comprehended the whole scene.

"Well, he's a reg'lar young scamp, and he played me a scurvy enough trick, or tried to do it; but upon my word you're a fine young feller, Arthur, and I would rather be you than—than some other people"—he glanced meaningly at his brother merchant, who had sunk down again in his chair and was lost in a gloomy abstraction. "You tell Fred from me that I bear no malice, and to prove it, there's a vacant clerkship in our West India house, and he can step into the berth if he likes. Climate ain't first class they tell me. A good many fevers and that sort of thing about, but beggars musn't be choosers. He can change his name and nobody will be a bit the wiser—north, south, west, any direction of the compass that pleases him will suit me." He laughed at his own humour.

Arthur went round the table and shook the jolly, fat old man by the hand.

"Thank you," he said, "I'll tell him." And then he walked out of the room. He was so sad of spirit that he was thankful for even this crumb of sympathy.

*(To be continued.)*

